# Diagnostic Accuracy of FOBT and Colorectal Cancer Genetic Testing: A Systematic Review & Meta-Analysis

**DOI:** 10.5334/aogh.2466

**Published:** 2019-05-15

**Authors:** Abdul Rahman Ramdzan, Muhammad Aklil Abd Rahim, Aznida Mohamad Zaki, Zuraidah Zaidun, Azmawati Mohammed Nawi

**Affiliations:** 1 Department of Community Health, Faculty of Medicine, Universiti Kebangsaan Malaysia, Kuala Lumpur, MY; 2 Department of Community and Family Medicine, Universiti Kebangsaan Malaysia, Sabah, MY

## Abstract

**Introduction:**

Colorectal cancer (CRC) is the second leading cause of cancer related death in the world after lung cancer. Early detection of CRC leads to improvement in cancer survival rate. In recent years, efforts have been made to discover a non-invasive screening marker of higher sensitivity and specificity. Fecal occult blood testing (FOBT) and genetic testing become alternative modalities to screen CRC in the population other than colonoscopy. The aim of this systematic review and meta-analysis is to determine the diagnostic accuracy, sensitivity and specificity of FOBT and genetic testing as screening tools in colorectal cancer.

**Methods:**

A literature search of PubMed, ScienceDirect, and Scopus was carried out. The search strategy was restricted to human subjects and studies are published in English. Data on sensitivity and specificity were extracted and pooled. Heterogeneity was assumed at significance level of p < 0.10 and was tested by chi squared. Degree of heterogeneity was quantified using the I^2^ statistic, and values of less than 25% is considered as homogenous. All analyses were performed using the software Meta-Disc.

**Results:**

A total of eleven studies were suitable for data synthesis and analysis. Five studies were analyzed for the accuracy of genetic testing, the pooled estimate for sensitivity and specificity were 71% (95% CI: 66, 75%) and 95% (95% CI: 93, 97%) respectively. Another group of studies which had been evaluated for the accuracy of FOBT, the pooled sensitivity was 31% (95% CI: 25, 38%) while the pooled specificity was 87% (95% CI: 86, 89%).

**Conclusions:**

FOBTs is recommended to use as population-based screening tools for colorectal cancer while genetic testing should be focusing on patients with moderate and high risk individuals.

## Introduction

Colorectal cancer (CRC) is the second most common cancer in the world [1]. It is the second leading cause of cancer related death worldwide after lung cancer. The American Cancer Society found that about one in 20 people in the US will develop colorectal cancer during their lifetime [2]. Early detection is the main key to improve cancer survival rate. Screening modalities for CRC have been initiated in several countries [3,4]. Clinical practice guidelines (CPG) recommend that average risk individuals begin regular screening for CRC at 50 years of age [5]. Colonoscopy, despite being the gold standard diagnostic tool, remains invasive with risk of perforation. Because of that, the population’s uptake of colonoscopy is low [6,7,8,9,10].

On the other hand, fecal occult blood testing (FOBT) is another modality to screen CRC among the population. FOBT using either guaiac-based (gFOBT) or immunological-based (iFOBT) methods are not invasive and more preferred [11,12,13]. It detects subtle blood loss in the gastrointestinal tract as a result of cancer bleeding. gFOBT detects heme while iFOBT detect globin [14].

In the past few years, there has been a drive to discover a more sensitive and specific tool in CRC screening. Genetic testing is one of the options. It can detect if members of certain families are prone to inherit any genetic link cancer, particularly first degree relatives [15,16]. Mutations inside MLH1, MSH2, MSH6 and PMS2, the genes involved in the DNA mismatch repair (MMR), boost the risk of developing CRC, especially Lynch syndrome (LS) [17,18]. Mutations in any of these genes impair the suitable repair of DNA replication errors that accumulate in the cells, promoting their aberrant proliferation leading to cancer. MLH1 and MSH2 germline mutations account for approximately 90% of mutations in families with LS [19,20].

However, there appear to be some contention in the literature regarding its utility. Some studies on cancer genetic testing have shown a favorable sensitivity and specificity for colorectal cancer and adenoma [21,22], whilst other studies have shown the opposite [23,24]. Otherwise, it is a less invasive blood based screening, thus highly preferable and accepted among patients.

According to Clinical Practice Guidelines, moderate- and high-risk groups will be referred to do a colonoscopy, however genetic testing can be offered [5]. All individuals whose family history is suggestive of a hereditary colorectal cancer syndrome should be referred to a clinical genetics service for genetic counselling, genetic risk assessment, and consideration of genetic testing to clarify the risk. These guidelines are yet to be used locally.

The aim of this systematic review and meta-analysis is to measure the accuracy of both FOBT and genetic testing as screening tools in colorectal cancer, specifically to identify the sensitivity and specificity of these modalities and their potentials as population-based screening tools.

## Methods

### Search Protocol

A systematic search was conducted (1 October 2018) on PubMed, ScienceDirect, and Scopus databases covering a period of five years (2013 to 2018) guided by Lui et al. (2013) [25]. Search was done for the relevant titles, abstracts, and keywords and adhered to the PICO strategy as stated in PRISMA checklist. The terms used for P (Problem or Population) was “colorectal cancer”, followed by terms for I (Intervention) which were “fecal occult blood test” OR “FOBT” OR “genetic testing”. The keywords used for O (Outcome) were “sensitivity” OR “specificity” OR “validity” OR “diagnostic accuracy”. For this review, there was no C (Comparison) terms in the search protocol. The respective MeSH terms for P, I, and O were also searched. Only English language literatures were searched and there was no restriction placed on location. Grey literatures and other sources were not searched.

### Study Selection

Studies were first screened by the title. After the first screening was done and the studies’ titles were deemed as having appropriate research question as this review, two reviewers were randomly allocated for each study for the screening of abstracts. Following this, the next phase of the article selection involved retrieval of the full articles for further scrutinization. Two reviewers, who were different from the initial reviewers, were allocated for each study for the perusal of the full text. Data extraction was conducted for the studies which had been accepted after review of the full article. Studies were only considered for this review and included if 1) it was an original article (not a review paper or commentary) and 2) the study had included a quantitative measurement of colorectal screening tool validity. The exclusion criteria were 1) inability to access the full article; 2) full article was not in English; and 3) the study had used combination of multiple tools or assessment criteria for screening of colorectal cancer (the study had used single or with combination of FOBT, colonoscopy and genetic testing).

### Assessment of Study Quality

Quality assessment for accepted studies was performed using the “Quality Assessment of Diagnostic Accuracy Studies” (QUADAS) tool. This tool was validated for the purpose of assessing study quality of studies included in diagnostic accuracy systematic review. It was developed by a consensus from nine experts in the field of diagnostics which took part in the Delphi procedure. QUADAS consists of 14 items of questions showed in Table 1 which cover bias (*items 3, 4, 5, 6, 7, 10, 11, 12 and 14*), variability (*items 1 and 2*), and the quality of reporting (*items 8, 9 and 13*) [26]. The validity of QUADAS by an evaluation by three reviewers rated 30 studies, with the range of agreements were very good; 85 to 91% [27].

**Table 1:** Items of assessment in QUADAS.

Items	Assessment	Questions

**Q1**	Adequate spectrum composition	Was the spectrum of patient’s representative of the patients who will receive the test in practice?
**Q2**	Clear description of selection criteria	Were selection criteria clearly described?
**Q3**	Adequate reference standard	Is the reference standard likely to correctly classify the target condition?
**Q4**	Absence of disease progression bias	Is the period between reference standard and index test short enough to be reasonably sure that the target condition did not change between the 2 tests?
**Q5**	Absence of partial verification bias	Did the whole sample or a random selection of the sample, receive verification using a reference standard of diagnosis?
**Q6**	Absence of differential verification bias	Did patients receive the same reference standard regardless of the index test result?
**Q7**	Absence of incorporation bias	Was the reference standard independent of the index test (i.e. the index test did not form part of the reference standard)?
**Q8**	Adequate description of the index test execution	Was the execution of the index test described in sufficient detail to permit replication of the test?
**Q9**	Adequate description of the reference test execution	Was the execution of the reference standard described in sufficient detail to permit its replication?
**Q10**	Absence of index test review bias	Were the index test results interpreted without knowledge of the results of the reference standard?
**Q11**	Absence of reference test review bias	Were the reference standard results interpreted without knowledge of the results of the index test?
**Q12**	Absence of clinical review bias	Were the same clinical data available when test results were interpreted as would be available when the test is used in practice?
**Q13**	Report of uninterpretable results	Were uninterpretable/intermediate test results reported?
**Q14**	Description of withdrawals	Were withdrawals from the study explained?

For the purpose of this review, item 12 was omitted because it was not relevant to the test and tool reviewed. Each study was randomly allocated to a reviewer for study quality assessment and subsequently the studies were also reviewed by another reviewer. The reviewers scored as ‘yes’, ‘no’, or ‘unknown’ for each items, and the level of agreement was stratified accordingly. Any disagreement was resolved by discussion and consensus.

### Data Extraction

A standardized table with relevant headings was used to extract data, namely the study design, population, sample size, gold standard for validity measurement, sensitivity, specificity, and other measure of screening tool validity. Studies were arranged according to year of publication.

### Statistical Analysis

Composite estimates of the sensitivity and specificity of each tool analyzed were tabulated in Forest Plots and compared with one another. Heterogeneity was assumed at significance level of p < 0.10 and was tested by chi squared. Degree of heterogeneity was quantified using the I^2^ statistic, and values of less than 25% was considered as homogenous. Arbitrary cut-off points of 50% and 75% were used to divide the studies into low, moderate, and high level of heterogeneity. All analyses were performed using the software Meta-Disc (version 1.4).

## Results

**Figure 1:**
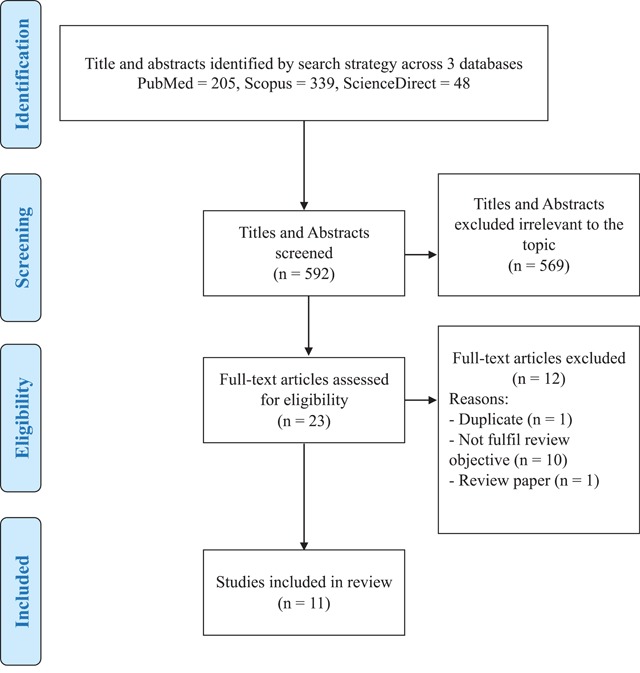
Flow Chart of Studies Selection.

Search results returned a total of 592 articles, of which 569 articles were excluded due to irrelevant title and abstracts during screening. Of these, 23 articles were eligible for review of full text. After full text article were extracted, 11 studies were included in review with six studies of FOBT suitable for meta-analysis (Figure 1).

Among the 11 studies included in diagnosing colorectal cancer, six studies examined the accuracy of FOBT test and another five studies examined genetic testing. For the purpose of quality assessment, we described the percentage of agreement by each QUADAS items according to the type of screening tools. The overall results of the quality assessment using QUADAS for each of the 11 studies reviewed, were presented in Table 2. An average, high percentage of agreements on both FOBT and genetic testing studies reviewed, it shows a good quality of diagnostic studies included in this review. Lower rate of agreement (FOBT 33.3%, genetic 40%) was observed on the withdrawal description among the studies.

**Table 2:** Assessment of methodological quality using QUADAS (n: 11).

No.	First Author (Year)	Screening Tool	Q1	Q2	Q3	Q4	Q5	Q6	Q7	Q8	Q9	Q10	Q11	Q13	Q14

**1.**	Yeasmin F (2013)	FOBT	Y	Y	Y	Y	Y	Y	Y	Y	Y	U	Y	N	N
**2.**	Lohsiriwat (2014)	FOBT	Y	Y	Y	Y	Y	Y	Y	Y	Y	U	U	U	N
**3.**	Redwood (2014)	FOBT	Y	Y	Y	Y	Y	Y	Y	Y	Y	Y	Y	U	Y
**4.**	Elsafi (2015)	FOBT	Y	Y	Y	Y	Y	Y	Y	Y	Y	Y	Y	Y	N
**5.**	Mario (2015)	FOBT	N	Y	Y	Y	N	N	Y	U	U	Y	N	N	N
**6.**	Shapiro (2017)	FOBT	Y	Y	Y	U	Y	Y	Y	Y	Y	Y	U	U	Y
**7.**	Dvorak (2014)	Genetic	Y	Y	Y	U	Y	Y	Y	Y	Y	Y	Y	Y	Y
**8.**	Amiot (2014)	Genetic	Y	Y	Y	N	Y	Y	Y	Y	Y	Y	Y	Y	N
**9.**	Johnson DH (2016)	Genetic	Y	Y	Y	N	Y	Y	Y	Y	Y	Y	Y	Y	N
**10.**	Kanth P (2016)	Genetic	Y	Y	Y	Y	Y	Y	Y	Y	Y	U	Y	Y	Y
**11.**	Xie (2018)	Genetic	Y	Y	Y	N	Y	Y	Y	Y	Y	Y	Y	Y	N
	% of agreement ‘yes’	FOBT	83.3	100	100	83.3	83.3	83.3	100	83.3	83.3	66.7	50	16.7	33.3
		Genetic	100	100	100	80	100	100	100	100	100	80	100	100	40

### Characteristic and Accuracy of Selected Studies

A total of eleven studies have been selected for this review. All those studies have been conducted at all regions across the world. Majority of the studies were carried out by United States of America and there were four studies done in Asia. Different study designs have been used in the selected studies, those are; prospective cohort, case-control, and cross-sectional with diverse objectives. Among the eleven studies, six studies assessed accuracy of FOBT (Table 3) and five on genetic testing (Table 4).

**Table 3:** Characteristic and accuracy of selected fecal occult blood test studies.

No	Author (Year)	Country	Study design	Study population	Index test	Reference test	Sample size	Sensitivity %	Specificity %	PPV %	NPV %	Accuracy %

**1.**	Shapiro et al. (2017) [29]	USA	Cross-sectional	Asymptomatic patients from clinics, aged 50–75 years.	HS-gFOBT	Not mentioned	1095	7.4	98.6	23.6		86.0*
**2.**	Mario et al. (2015) [30]	Brazil	Cross-sectional	Asymptomatic patients, aged ≥ 50 years.	gFOBT & flexible RSS	Colonoscopy	102	30.0	92.4	30.0*	5.0*	86.3*
**3.**	Elsafi et al. (2015) [31]	Saudi Arabia	Cohort	Asymptomatic patients aged 50–74 years old from 2 hospitals and confirmed CRC patients.	gFOBT		Cases 257 Control 20	50.0	77.9	3.5	99.0	71.8*
**4.**	Lohsiriwat et al. (2014) [32]	Thailand	Case-control	Histologically proven adenocarcinoma of the colon and rectum patients and individuals with normal colonoscopic findings.	FOBT	Colonoscopy	Cases 96 Control 101	41.0	97.0	93.0	63.0	70.0
**5.**	Redwood et al. (2014) [33]	USA	Cross-sectional	Asymptomatic adults aged ≥ 40 years.	gFOBT	Colonoscopy	304	28.5	75.7	10.6	91.3	71.4*
**6.**	Yeasmin et al. (2013) [34]	Bangladesh	Cross-sectional	Patients suspected to have occult bleeding.	gFOBT	Colonoscopy	110	75.0	21.6	7.0	91.7	25.5*

FOBT- Fecal occult blood test. gFOBT- Guaiac fecal occult blood test. HG-gFOBT- High-sensitivity guaiac fecal occult blood test. RSS- Recto sigmoidoscopy. PPV- Positive Predictive Value. NPV- Negative Predictive Value. * indirectly calculated from data.

**Table 4:** Characteristic and accuracy of genetic testing.

No	Author (Year)	Country	Study design	Study population	Index Test	Reference Test	Sample size	Sensitivity %	Specificity %	PPV %	NPV %	Accuracy %

**1.**	Xie (2018) [35]	China	Case control	Patients aged >18 years old with histologically confirmed mCRC.	mSEPT9	Colonoscopy	123 Cases 125 Controls	61.0	89.0	89*	62.8*	73.4*
**2.**	Johnson (2016) [36]	USA	Case control	Advanced CRC patients.	BMP3	Histology	17 Cases 12 Controls	76.0	92.0	92.9*	73.3*	82.8*
**3.**	Kanth (2016) [37]	USA	Case control	Asymptomatic patients from 2 medical centers, aged age 45–75.	BRAF	Colonoscopy	41 Cases 20 Controls	94.0	72.0	79.5*	90.9*	83.6*
**4.**	Dvorak (2014) [38]	Australia	Cross-sectional	Colorectal and Papillary thyroid cancer patients.	BRAF	Histology	352	98.6	99.1	98.6	99.1	98.9*
**5.**	Amiot (2014) [39]	France	Case control	Asymptomatic patients from a teaching hospital.	Wif-1 Gene	Colonoscopy	90 Cases 157 Controls	33.0	99.0	96.9*	70.7*	74.1*

PPV- Positive Predictive Value. NPV- Negative Predictive Value. * indirectly calculated from data.

### FOBT

A total of six studies assessed on accuracy of FOBTs. All of the studies evaluated Guaiac FOBTs. Majority of the studies were done cross-sectional, while there are two studies done as cohort and case-control. The study population were mostly asymptomatic adults aged 50 years and above. Diagnostic accuracy of FOBT ranged from 25.5% to 86.3% with sensitivity and specificity ranged from 7.4%–75.0% and 21.6%–98.6%, respectively.

### Genetic Testing

These five articles were studies from 2014–2018 from USA, China, Australia, and France. All this study used high risk group as their population. In the other hand, two studies used BRAF as index test, and others used mSEPT9, BMP3, and Wif-1 Gene, respectively. A total of 937 patient data were pooled from the five articles. Of these, 302 patients had a confirmed diagnosis of colorectal cancer on colonoscopy and histology. Using genetic testing, 302 (32.23%) of participants were identified as true positives, 23 (2.45%) as false positives, 486 (51.87%) as true negatives and 126 (13.45%) as false negatives. The accuracy of genetic testing is as showed in Table 4. The sensitivity and specificity of genetic testing for diagnosing colorectal cancer was range 33–98 and 72–99 respectively. The diagnostic accuracy of genetic testing for diagnosing colorectal cancer was range 73.4–98.9.

### Meta-Analysis

**Figure 2:**
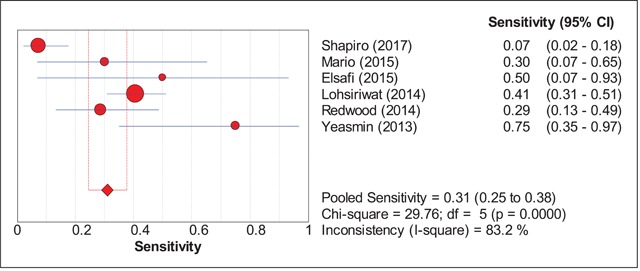
Pooled Sensitivity FOBT.

**Figure 3:**
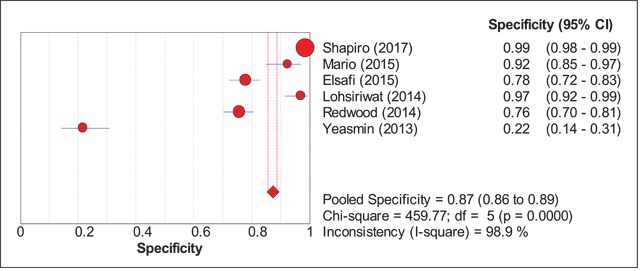
Pooled Specificity FOBT.

The sensitivity and specificity figures of all the studies were pooled to obtain an estimate of the diagnostic accuracy of FOBT in detecting colorectal cancer. There were six studies which had evaluated the accuracy of chemical or guaiac FOBT to detect colorectal cancer (Shapiro et al. [2017]; Mario et al. [2015]; Elsafi et al. [2015]; Lohsiriwat et al. [2014]; Redwood et al. [2014]; Yeasmin et al. [2013]). For this group, the pooled sensitivity was 31% (95% CI: 25, 38%) while the pooled specificity was 87% (95% CI: 86, 89%) (Figures 2 and 3). For analyses within the subgroups, significant heterogeneity was observed, ranging from 83.2% to 98.9% of heterogeneity as quantified by I-square test. All findings are summarized in Table 5.

**Table 5:** Summary Statistics for the Diagnostic Accuracy of FOBT.

Sub-groups	Number of Studies	P-value^a^	I^2^ (%)^b^	Sensitivity % (95% CI)	Specificity % (95% CI)

FOBT	6	<0.001	83.2–98.9	31 (25, 38)	87 (86, 89)

^a^ P-value for heterogeneity (chi-square) for both sensitivity and specificity analyses. ^b^ I^2^ statistics for heterogeneity quantification for both sensitivity and specificity analyses.

## Discussion

### Principal Findings

This review had found 11 studies that addressed the diagnostic accuracy of genetic testing modalities and FOBT to detect colorectal cancer in the last 5 years. Chemical or guaiac FOBT has been used as a screening tool for colorectal cancer in symptomatic persons mostly due to its affordable cost. Hirai et al. (2016) reported pooled sensitivity & specificity of FOBTs for CRC detection in the proximal colon were 71.2% (95% CI 61.3–79.4%) and 93.6% (95% CI 90.7–95.7%) respectively. Both gFOBT and iFOBT showed significantly lower sensitivity but comparable specificity for the detection of proximally located CRC compared with distal CRC [28].

Moving into the era of precision medicine, genetic testing is fast gaining traction as a screening tool for those without symptoms but having strong family history or having known family members with genetic mutations associated with colorectal cancer. However, its high cost and the need for specialized service is hampering efforts to introduce the tool in resource-modest countries, including developing countries. Present study shows that genetic testing modalities had relatively better diagnostic accuracy as compared to FOBT in detecting colorectal cancer. Genetic testing was shown to be superior to FOBT in diagnostic accuracy range 73.4–98.9 versus diagnostic accuracy of FOBT range 25.5–86.3. However, most of the genetic testing studies were conducted among the high-risk group or confirmed CRC patients which may affect the outcome.

Since genetic testing has a superior diagnostic accuracy compared to FOBT, it can be suggested to be included in the CPG for screening of asymptomatic moderate- and high-risk people, provided that a prevalence study of the genetic variant responsible for CRC is available locally and its cost effectiveness analysis is done.

### Analysis of Heterogeneity

Our results also showed that there was significant heterogeneity in the analysis in the subgroup. There are multiple possibilities as to why these heterogeneities came about. First, the studies were conducted in different populations and localities. The intrinsic differences between populations also extend into differences between socio-economic status, cultures, and quality of medical services. These dissimilarities may have affected the accuracy of the tools used. Since the number of studies under FOBT group (six studies) were already in single digits, it was not feasible to further sub-divide the studies into sub-groups. Doing so may improve the homogeneity between studies, but pooling accuracy estimates from two or three studies may not generate results that are generalizable to the bigger populations.

On the other hand, there were multiple genes being tested in genetic testing. Each of the five studies which had assessed genetic testing modalities had a different method of detecting the affected gene for colorectal cancer. Although these studies were classified together under “genetic testing”, there existed a measure of heterogeneity in their methods and specific marker that each study had appraised.

### Strengths and Weaknesses

Our review had summarized the accuracy of genetic testing modalities and FOBT in detecting colorectal cancer from studies conducted in the last five years. This means that the results of this review are up-to-date with current evidence and the data for diagnostic accuracy of assessed tools can be incorporated into devising a health policy or guidelines in colorectal cancer prevention and management.

However, there are also a few notable weaknesses. We only searched English literatures and hence a degree of bias is expected. Pooling accuracy estimates taken only from the most recent studies may also introduce a significant bias. Apart from that, small number of studies included also made further sub-group analysis impractical and thus heterogeneity was not able to be fully explored and rectified. There were only five studies on genetic testing which studied different genes. Therefore, pool analysis for genetic testing cannot be carried out.

Lastly, we did not address other factors related to the implementation of the tools, such as the specific type of genetic testing modality, the patient clinical history, and the colorectal cancer histologic manifestations. We presume that the results were useful to suggest the level of diagnostic accuracy of the tools assessed, but is limited in robustness in view of the limitations mentioned.

## Conclusion

The sensitivity and specificity of FOBTs are 31% (95% CI; 25, 38) and 87% (95% CI; 86, 89) respectively. The accuracy of genetic testing is inconclusive due to limited number of studies. For future reviews, it is recommended to omit time limitation and to include grey literatures and more evidence of FOBT and genetic testing accuracy. To propose, genetic testing should be considered as a screening tool (such as MHL1, MSH2, MSH6, Epcam, Braf v600e) for all moderate- and high-risk asymptomatic people, after genetic variant prevalence and cost effectiveness study provide further favorable evidence. It is suggested to use FOBT as population-based screening instead of opportunistic screening.
